# Supplementary material for: Angelica sinensis polysaccharide as potential protectants against recurrent spontaneous abortion: focus on autophagy regulation
Source: Front Med (Lausanne). 2025 Jan 15;12:1522503. doi: 10.3389/fmed.2025.1522503 (PMC11774876; doi:10.3389/fmed.2025.1522503)
Supplement: Supplementary material S1 — The main instruments used during the LC-MS process, along with their models/specifications and manufacturers. [file Supplementary_file_1.zip › Metabolomics sequencing data FC1.2/8.代谢通路分析/Exposure_Ctrl/all/KEGG/mmu00860.html]

KEGG PATHWAY: Porphyrin metabolism - Mus musculus (house mouse)


# Porphyrin metabolism - Mus musculus (house mouse)


[
Pathway menu
| Organism menu
| Pathway entry
| Show description
| Download
| Help
]

Porphyrins are a group of chemical compounds with the backbone ring structure consisting of four linked pyrrole units. Metal complexes of porphyrins, as well as those of corrinoids with the one-carbon shorter ring structure, play important biological roles. They include iron-containing heme, magnesium-containing chlorophyll, nickel-containing coenzyme F430 and cobalt-containing cobamide (such as vitamin B12 coenzyme) as shown in this map.


##### Option

Scale:


100%

Image resolution:


 High

##### Search

##### ID search

##### Color

##### Module

Complete only
Including 1 block missing
Including any incomplete

- Pathway modules
  - Metabolism of cofactors and vitamins
    - Cofactor and vitamin metabolism
      - M00846
        Siroheme biosynthesis
      - M00868
        Heme biosynthesis, animals and fungi
      - M00121
        Heme biosynthesis, plants and bacteria
      - M00926
        Heme biosynthesis, bacteria
      - M00847
        Heme biosynthesis, archaea
      - M00924
        Cobalamin biosynthesis, anaerobic
      - M00925
        Cobalamin biosynthesis, aerobic
      - M00122
        Cobalamin biosynthesis
      - M00836
        Coenzyme F430 biosynthesis


KGML

Image (png) file 1x
